# Misdiagnosed cartilaginous PCL avulsion in young children

**DOI:** 10.1051/sicotj/2021052

**Published:** 2021-11-19

**Authors:** Romain Pacull, Florian Bourbotte-Salmon, Margaux Buffe-Lidove, Nicolas Cance, Franck Chotel

**Affiliations:** 1 Service de Chirurgie Orthopédique et Traumatologie, Hôpital Edouard Herriot 5 Place d’Arsonval 69003 Lyon France; 2 Service de Médecine Physique et de Réadaptation, Hôpital d’instruction des Armées Desgenettes 108 Boulevard Pinel 69003 Lyon France; 3 Service de Chirurgie Orthopédique Pédiatrique, Hôpital Femme Mère Enfant de Lyon Groupement Hospitalier Est, 59 Boulevard Pinel 69500 Bron France; 4 Université Claude Benard Lyon I 43 Bd du 11 Novembre 1918 69100 Villeurbanne France

**Keywords:** Posterior cruciate ligament, Cartilaginous femoral avulsion, Children, MRI, Bony, Misdiagnosis

## Abstract

Posterior Cruciate Ligaments injuries are rare in children and usually due to bony avulsion fractures or midsubstance tears. This study focused on cartilaginous avulsions initially misdiagnosed despite of MRI assessment. Two 6-year-old boys had cartilaginous avulsion fracture injury at the femoral attachment of the PCL. One had associated medial meniscal lesion and was reinserted. The other conducted to non-union. MRI second lecture reveals an original description with nail-biting sign on cartilage surface of anterior notch, and a close PCL angle without anterior tibial translation. No bone bruise was associated. Similarly, to ACL cartilaginous tibial avulsions, PCL cartilaginous femoral avulsions are underdiagnosed. When knee hemarthrosis occurs under the age of nine, clinician and radiologist should be aware that cartilaginous avulsion of ACL and PCL also could be the main pattern of lesion.

## Introduction

Despite an increasing number of Anterior Cruciate Ligament (ACL) ruptures in children and adolescents, Posterior cruciate ligament (PCL) lesions are still very rare. The great majority of literature about PCL injury in children are cases reported. In young children under 9 years, cartilaginous avulsions of the tibial insertion of ACL have been recently reported as a new entity frequently misdiagnosed [1]. Similarly, purely cartilaginous avulsions are a modality of PCL lesions in young children. The authors reported two cases of misdiagnosed cartilaginous avulsion of the femoral insertion of PCL despite Magnetic resonance imaging (MRI) assessment. The analysis focuses on MRI signs to identify to allow proper management.

## Methods

- A six-year-old boy was admitted to the emergency department for a right knee injury after a trampoline accident. The acute physical examination only revealed hemarthrosis, diffuse knee pain, and antalgic flexion. Knee radiographs were considered as normal. The patient was prescribed symptomatic treatment and MRI. One month later, the MRI highlighted an isolated and unexplained persistent joint effusion. The clinical outcomes were satisfying; a functional range of motion was recovered (10-0-125). The physical examination in the tertiary referral center for the pediatric knee department did highlight a posterior drawer sign and an anterior tibial tuberosity erasing. Stress X-rays in the posterior manual drawer confirmed the PCL insufficiency (cf. Figure 1). A new MRI was performed by a pediatric radiologist aware of the possibility of a PCL injury and gave the final diagnosis: a purely cartilaginous avulsion of PCL on its femoral attachment and a radial lesion of the posterior segment of the medial meniscus (cf. Figure 2). Meniscal tear, important laxity due to displaced avulsion, and poor clinical outcomes improvement motivated arthroscopy-assisted ligament reinsertion, and a meniscal repair performed three months after the traumatism. Femoral PCL attachment was fixed thanks to meniscal suture, ligament pull-out suture and use of anchors. Despite specific rehabilitation protocol, the evolution was limited by a major knee mobility restriction (range of mobility: 0-20-70), and an arthroscopic arthrolysis was performed six months after the reinsertion surgery Figure 3. After 3 years of follow-up, the child returned to its pre-injury level of activity. The side-to-side difference in posterior translation as measured with stress radiographs was 2 mm; the mean Lysholm score was 95 points. The patient was classified A in the International Knee Documentation Committee form.
  **Figure 1:**
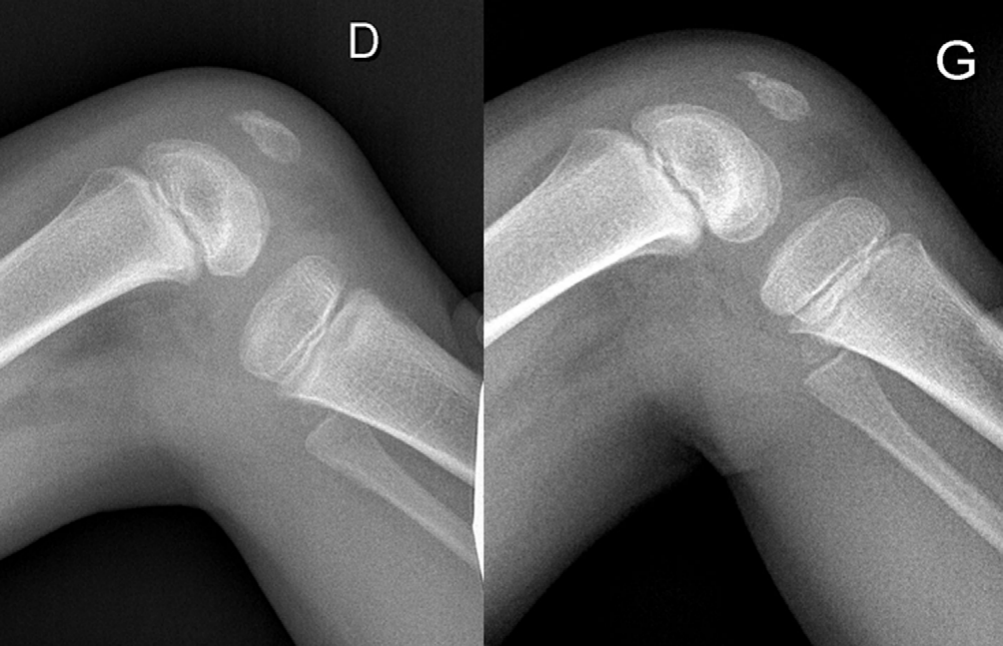
Case 1. Posterior drawer sign with more 10 mm on stress radiographs of the right knee. No bony avulsion was noticed.
  **Figure 2:**
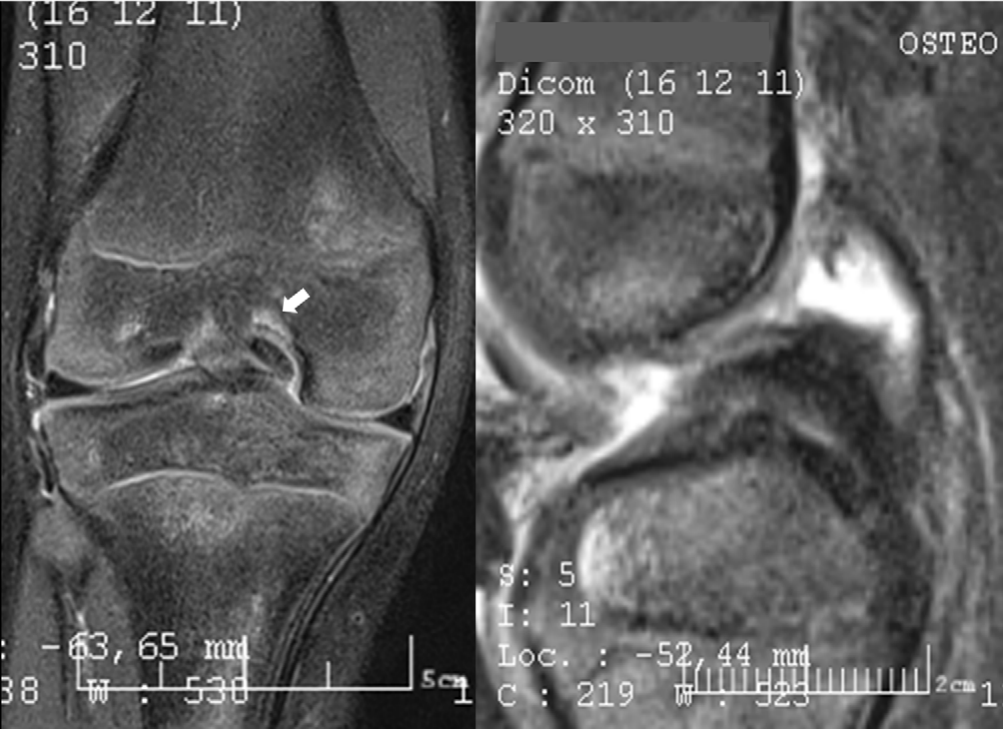
Case 1. MRI aspect after second assessment reveals cartilaginous nodule in the intercondylar notch with femoral oedema in coronal plane (left) and abnormal thickening of the proximal part of the PCL (right).
  **Figure 3:**
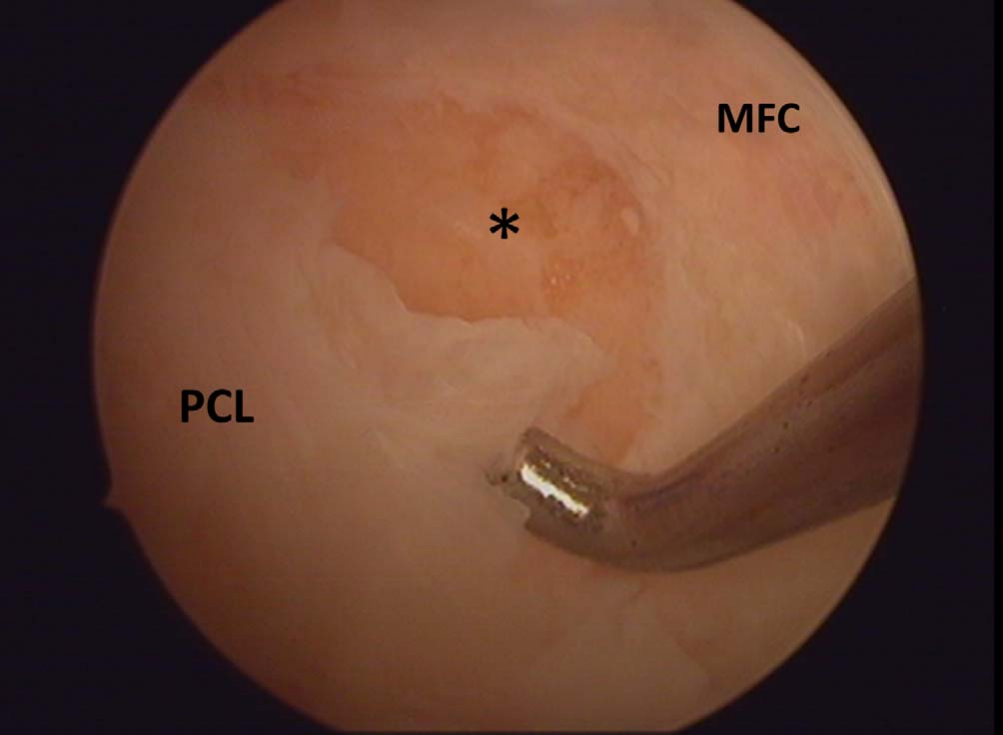
Arthroscopic view of the PCL’s cartilaginous femoral defect after debridement. MFC = Medial Femoral Condyle.
- The second patient, also six years old boy, suffered a torsional right knee trauma after water toboggan bad reception. Despite immediate and severe joint effusion, the patient was initially diagnosed with a medial collateral ligament (MCL) sprain in the hospital emergency room. He had 45 days of immobilization in a removable knee splint. The MRI performed in the following month was interpreted as partial disinsertion of the MCL with moderate joint effusion. Because of persistent limping and a new trauma with functional disability 1 year after the trauma, a new appointment in a specialized clinic was taken by parents. Physical examination showed no joint effusion but a lack of physiological hyperextension. A new X-rays showed a suspect ossification in the intercondylar notch area (cf. Figures 4 and 5). New MRI confirmed that the ossification came from the initial cartilaginous avulsion and confirmed a non-union. Arthroscopic reinsertion of the osteochondral fragment was performed 9 months after the trauma, and a good result was achieved 1.5 years later with a return to preinjury level of activity. The side-to-side difference in posterior translation as measured with stress radiographs was 4 mm; the mean Lysholm score was 92 points, and the patient was classified B in the International Knee Documentation Committee form.
  **Figure 4:**
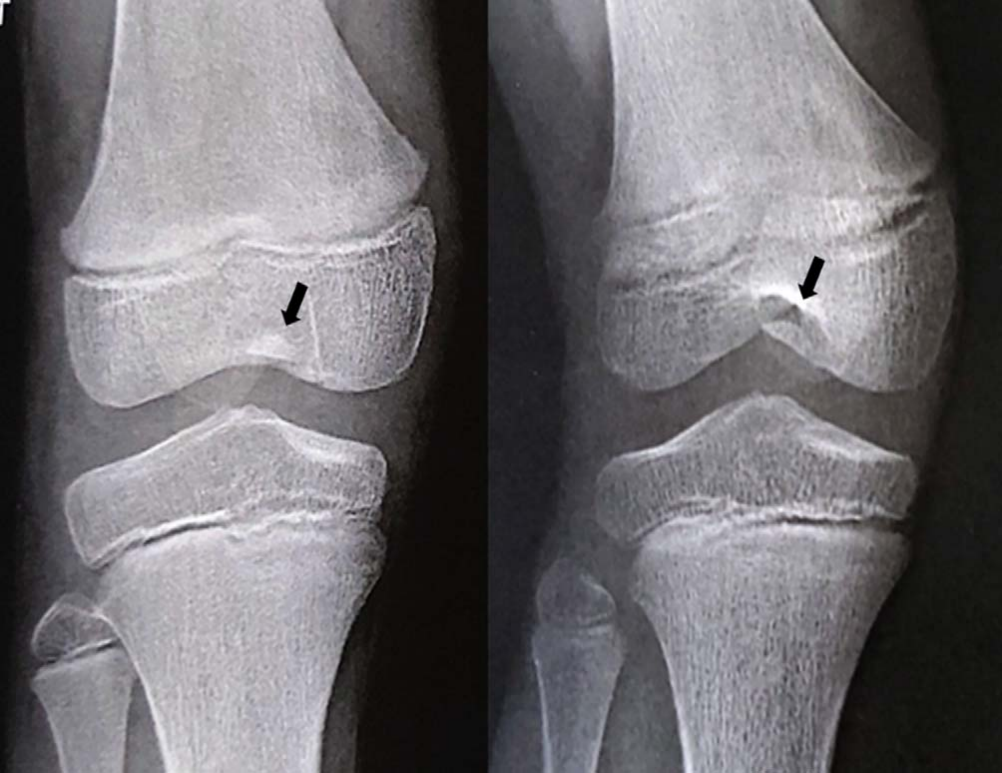
Case 2. AP and oblique views with ossification in the intercondylar notch area, one year following the initial trauma.
  **Figure 5:**
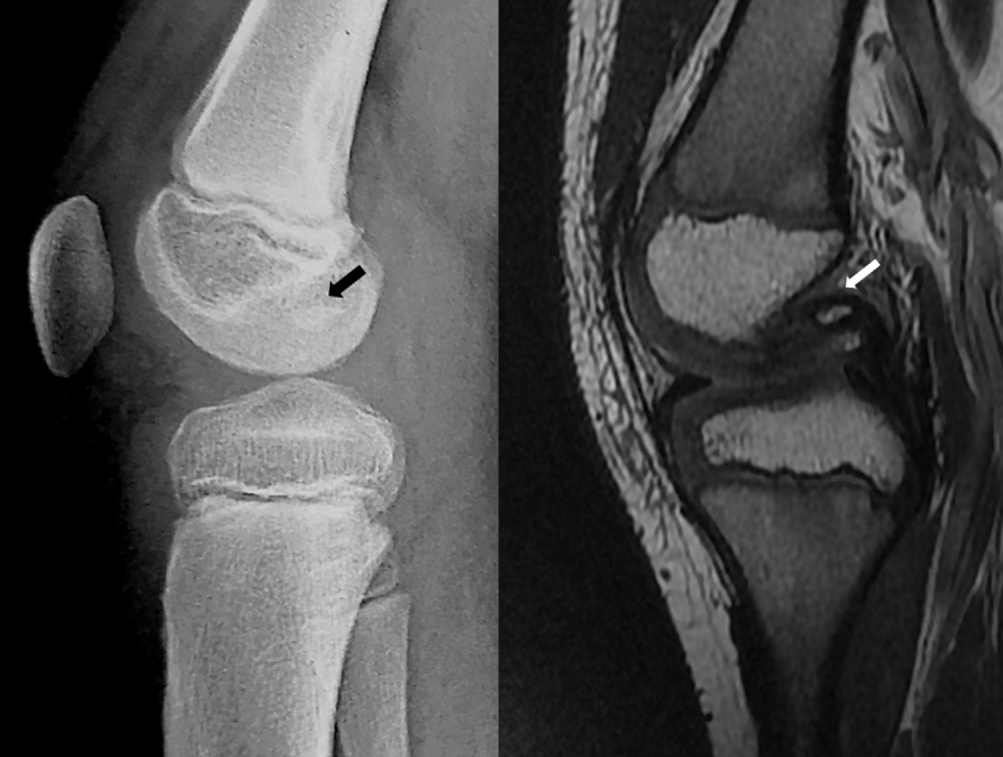
Case 2. Lateral view and sagittal sequences of second MRI showing ossification of the cartilaginous PCL avulsion on the femur.

For both children, joint effusion, flexion contracture, and pain did limit the clinical examination in the emergency room, and the lesion was not suspected. According to recommendations, the hemarthrosis associated with the absence of bony avulsion on standard Antero-posterior (AP) and lateral X-rays led to MRI prescription [2, 3]. In both cases, the MRI was wrongly interpreted. A PCL injury is so rare in children that neither clinicians nor radiologists did suspect it. A second lecture with senior pediatric orthopedic surgeon of the first MRI allows identifying specific signs of cartilaginous PCL avulsion compared to traditional direct or indirect signs reported in adults PCL injuries [4].

**Figure 6:**
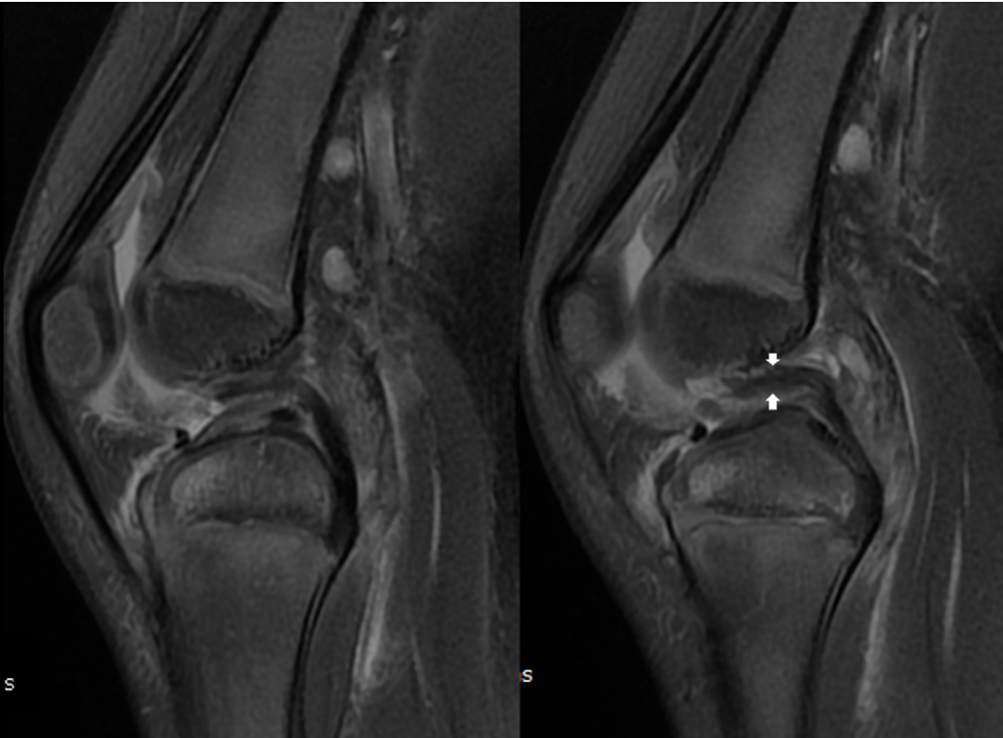
Case 2. First MRI with DP FS and sagittal sequences. Thickening of the proximal part of PCL (white arrows) with decreased PCL angle (right) and intact ACL (left).

**Figure 7:**
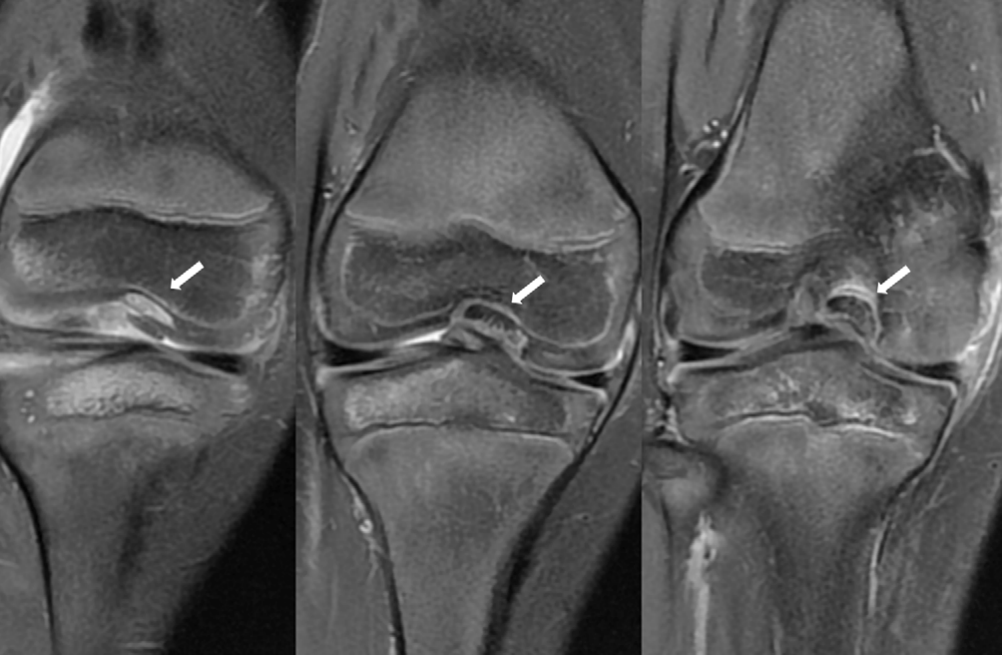
Case 2. First MRI with DP FS and coronal sequences. Cartilaginous nodule and “nail-biting” aspect in the medial part of the intercondylar notch (white arrows) was misdiagnosed.

For patient 1, in T2 weighted Fat Suppressed (T2 FS) MRI sequences, there was thickening of the whole PCL associated with a decreased PCL angle in sagittal view. In the coronal view, a notch nodule aspect lying on the medial condyle was found to benefit from hindsight. A “Nail-biting” aspect was noticed in posterior images. No significant sign of bone bruise or posterior tibial translation was visible on the same sequence. For patient 2, the MRI signal sequence performed was DP FS and T1, but no T2 or gradient echo sequence was carried out. T1’s very bad signal did not allow any diagnosis. The Density Proton Fat Suppressed (DP FS) sequence with sagittal images reveals abnormal thickening of the proximal part of the ligament that appear as a continuous structure without redundant or retracted aspect (cf. Figure 6). The PCL angle seems decreased without anterior tibial translation or ACL disruption (cf. Figure 6). Coronal images reveals a nodule or “Nail-biting” aspect on the chondral structure on the PCL femoral attachment (cf. Figure 7). No significant bone bruise was noticed on DP FS Sequence in the sagittal and coronal plane.

## Discussion

The prevalence of acute hemarthrosis after a knee injury is increasing with age. It remains rare in children under nine: only eight cases out of 138 (aged from 1 to 15 years) were reported in Vähäsarja et al.’s arthroscopic study [5]. This observation led Sweden to set 9 years old as the age limit for MRI exploration of the acute knee with hemarthrosis in their recommendations [2, 5]. These recommendations were not appropriate for the two cases reported here. The indication of MRI exploring acute knee hemarthrosis should not be limited by age [6]. But even with MRI acutely performed in the two cases, the diagnosis was missed by misinterpretation of signs and low suspicion of ACL or PCL lesion considering the age of the children.

It is traditionally taught to think about bony tibial avulsion of ACL (old “tibial spine”) or bony femoral avulsion of PCL in immature skeletal patient and midsubstance tear after closing growth plate. A new entity has been clearly identified under 9 years of age concerning purely cartilaginous ACL avulsion [1, 7]. Specific MRI signs and management have been precise [1]. The present study and literature analysis add major data about the “symmetric” entity on the PCL side in a similar context.

When including the two cases reported here and an extensive and recent literature review focused on PCL lesion in children, only 14 patients were under the age of nine (cf. Table 1). Among these patients, 10 had a cartilaginous femoral PCL lesion, 1 had a bony femoral avulsion, and 3 cases had midsubstance rupture. Of course, there is a bias of publication of rare cases. But, similarly to ACL cartilaginous avulsions, this entity could be the main mode of rupture of PCL in very young children.

**Table 1:** Recent literature review of cases reported PCL injuries in children under the age of nine.

Year	Authors	Number of patients	Age	Accident/mechanism	Kind of injury	Therapeutic strategy
1979	Clanton et al. [8]	2	6/9	Playing on a merry-go-round and falling of moving car / NA	Femoral cartilaginous avulsion for both + ACL tibial cartilaginous avulsion, LCL and arcuate ligament torn, MM detached (only for the second child)	Arthrotomy with ACL and PCL osteosuture; posterior capsule, LCL, arcuate complex all reefed; medial meniscectomy
1980	Sanders et al. [9]	2	6/8	For both Playing on a merry-go-round / Hyperextension	For both femoral cartilaginous avulsion	Arthrotomy with osteosuture
1989	Frank and Strother [10]	1	7	Ski accident / NA	Femoral Cartilaginous avulsion	Misdiagnosis Delayed surgery
1997	Lobenhoffer et al. [11]	1	3	NA	Femoral cartilaginous avulsion	Transosseous suture
2003	MacDonald et al. [12]	1	6	Trampoline accident	Intrasubstance tear	Surgery 5 years after injury
2006	Hesse et al. [13]	1	9	Fall from high elevation	Femoral bony avulsion + rupture of popliteal artery	Saphenous vein bypass graft then transosseous femoral sutures
2007	Shen et al. [14]	1	5	Direct blow on knee in hypertextension	Femoral cartilaginous avulsion + bucket-handle tear of the PHMM	Arthrotomic reinsertion + meniscus suture
2007	Anderson and Anderson [15]	1	8	Hyperextension	Intrasubstance + postero-lateral ligament tears	Surgery 5 years after injury, *STG* autograft + postero-lateral ligament reconstruction
2011	Scott and Murray [16]	1	4	Hyperflexion	Intrasubstance tear	Physiotherapy
2012	Kocher et al. [17]	1 out of 26	4	NA	Femoral cartilaginous avulsion	A wide variety and combination of surgical techniques arthroscopic or not.
2021	Bourbotte	2	6/6	Trampoline accident and water toboggan bad reception/knee torsion	Femoral cartilaginous avulsion for both + PHMM lesion for one	Arthroscopic fixation with anchors and in-out meniscal repair

NA: not available, ACL: anterior cruciate ligament, PCL: posterior cruciate ligament, LCL: lateral collateral ligament, MCL: medial collateral ligament, STG: semitendinosus-gracilis, MM: medial meniscus, PHMM: posterior horn of the medial meniscus.

Unlike ACL, where cartilaginous avulsion is located on the tibial, the weak attachment could be the femoral side for PCL in children. Bony tibial avulsion could be seen in older patients due to flexion trauma or direct blow to the anterior tibia. This distribution could also be due to the energy of the trauma and the injury mechanism. Young children usually have domestic accidents or low-energy trauma on nearly extended knees as reported in our cases. The low energy trauma may also explain that PCL lesion is often isolated. However, the first patient in the present study had a radial medial meniscus tear that needed repair. The presence of a meniscal lesion without malformation associated in a very young patient should bring central pivot associated lesion to mind, either ACL or PCL.

Despite the cases reported, cartilaginous PCL avulsion is probably an underdiagnosed lesion. Clinical exam is of low value in the acute phase, especially in the youngest. The presence of hemarthrosis increases the difficulty of diagnosing. The latter, associated with normal X-rays, require at least a new clinical assessment at a distance. In our two cases, the diagnostic was clinically suspect before MRI confirmation and the second lecture of the first images. Some authors suggest practicing ultrasound as an alternative for PCL tears diagnosis, but radiologist should be aware what he is looking at and be well trained.

Finally, MRI is the choice exam, but attention must be paid to the youngest to check the tibial ACL and femoral PCL cartilaginous insertions. Many factors may explain misdiagnosed lesions in the two reported cases. Wessel mentioned that the accurate evaluation of chondral or osteochondral fractures was somewhat problematic; it would require a gradient echo sequence, but often overlooked [6]. Eiskjaer et al. related one false positive and five false-negative about 31 cases [4]. Such signal sequence was not used in patient one and two. The apparent ligament thickening on sagittal T2 weighted images with cut-off value was established for adults, but there is no data for children. Another indirect sign often missing in skeletally immature patients, and especially in younger, is a bone bruise. None of both cases had such a sign. This could be due to a shock-absorbing function of the chondoepiphysis or the physis. Variation of PCL angle can be another sign of pivot lesion, but there are some limits of angle variations with age [18].

As reported for cartilaginous tibial avulsion of ACL, the cartilaginous avulsion fracture of the femoral attachment of the PCL in children must be suspected after knee hemarthrosis under the age of nine. Normal X-ray in this context should orient to new clinical assessment, MRI, and repeated X-rays when diagnosis is delayed. MRI gives valuable information as long as it is performed by an experienced and clinically oriented radiologist. Specific direct or indirect signs of PCL femoral avulsion are nail-biting sign on the cartilage surface of the anterior notch and close PCL angle without anterior tibial translation. Few weeks after misdiagnosis, ossification of the avulsed fragment occurs, and new X-rays can help diagnose and manage before non-union.

## Conflicts of interest

The authors declare that there is no conflict of interest.

## Funding

This research did not receive any specific funding.

## Ethical approval

Ethical approval was not required.

## Informed consent

Retrospective study. The 2 patients family’s gave consent for publication.

## Authors contribution

Contribution in data analyse and writing manuscript for Romain Pacull and Florian Bourbotte-salmon. Contribution in writing manuscript for Margaux Buffe-Lidove and Nicolas Cance. Contributions to conception & design, interpretation of data and writing manuscript and submission for Franck Chotel.
